# A Rare Case of Pseudomonas luteola Bacteremia Complicated by Cerebral Venous Sinus Thrombosis

**DOI:** 10.7759/cureus.62403

**Published:** 2024-06-14

**Authors:** Ernestine Faye S Tan, Sindhu C Pokhriyal, Muthanna Mohammed Hasan Al-Ghuraibawi, Yohannes D Gelan, Isaac Solaimanzadeh

**Affiliations:** 1 Internal Medicine, Interfaith Medical Center, Brooklyn, USA

**Keywords:** chryseomonas luteola, chryseomonas, bacterial meninigitis, neurology, venous sinus thrombosis (vst), cerebral venous sinus thrombosis (cvst), meningitis, pseudomonas, pseudomonas luteola

## Abstract

*Pseudomonas luteola (P. luteola)*, or *Chryseomonas luteola*, is an organism rarely reported as a human pathogen of concern. Commonly missed due to its rarity, emerging literature has shown its potential for pathogenicity; therefore, increased vigilance must always be observed in dealing with these bacteria, especially in immunocompromised patients and those with indwelling catheters, foreign bodies, and prosthetic implants. We present a case of a patient who came in for a persistent headache, found to have meningitis, and ended up with cerebral venous sinus thrombosis (CSVT) on further investigation. Interestingly, the patient did not have any other medical conditions, nor did he have any indwelling catheters or foreign bodies. After an extensive literature review, we report the first case of CSVT caused by *P. luteola* meningitis in an immunocompetent patient, and we aim to shed light on the diagnosis and treatment approach in this unusual case.

## Introduction

*Pseudomonas luteola *(*P. luteola*), alternatively termed *Chryseomonas luteola*, is an organism rarely reported in the literature as a human pathogen [1]. Since 1980, less than 30 cases have been reported worldwide, and less than five cases have been seen in immunocompetent patients[2].

Several case reports associate this organism with patients in immunocompromised states or in immunocompetent patients who have indwelling foreign bodies, such as prosthetic cardiac valves, dialysis catheters, intracranial prosthesis, and intravascular catheters [3]. Cases are mostly nosocomial in nature, although rare case reports have mentioned community-acquired infections^ ^[2,4].

*P. luteola* is a strictly aerobic, non-spore-forming, Gram-negative, motile rod. It grows on MacConkey agar, producing yellow-pigmented colonies that help distinguish it from other *Pseudomonas* [3]. Biochemically, they are non-lactose fermenters, oxidase-negative, and catalase-positive[4]. Case reports have associated this pathogen with septicemia, peritonitis, bacteremia, meningitis, systemic lupus erythematosus, and endocarditis[3,4,5]. Clinical isolates of *P. luteola* in several studies have demonstrated variable sensitivity and resistance patterns to different antibiotics [3,4,5].

Due to the paucity of literature regarding this organism, we present an unusual case of *P. luteola* bacteremia in a 46-year-old immunocompetent adult who developed acute meningitis with no identifiable risk factors. To our knowledge, this is the first case of *P. luteola* bacteremia seen in a patient found to have bacterial meningitis that eventually developed cerebral venous sinus thrombosis. 

The identification of a rare organism including *P. luteola* in fatal cases, such as meningitis, requires increased vigilance and awareness, especially in the critically ill, immunocompromised, and those who have foreign materials in their body. This report aims to increase awareness regarding this organism, to help with early diagnosis of infections, understand the pathologic complications, and avoid further morbidity and mortality.

## Case presentation

This is a case of a 46-year-old male from the Dominican Republic who worked as a freelance contractor in construction and environmental sanitation. The patient reported no significant past medical or surgical history and no recent travel within 12 months. Upon arrival at the emergency room, the patient complained of a two-month history of intermittent bifrontal and occipital dull and non-pulsating headaches. This was accompanied by nausea, generalized body weakness, and photophobia, for which he previously visited three other institutions, all of which diagnosed him with migraine headaches. 

Interval history revealed persistence of the headache, not relieved by his usual prescription medications like acetaminophen and ibuprofen. This was associated with projectile vomiting for two days. The criteria for systemic inflammatory response syndrome (SIRS) were fulfilled on admission coupled with elevation of lactate levels; hence, the patient was started on meropenem and vancomycin. Further workup revealed a urinary tract infection, which was considered the trigger of sepsis. The patient had persistent hyponatremia, with sodium levels ranging between 125 and 128 mg/dL, which persisted despite clinical improvement. Co-syntropin test was done, revealing adrenal insufficiency, and hydrocortisone was started. On the third hospital day, the blood culture grew Methicillin-sensitive* S. aureus* (MSSA) while the urine cultures were negative. Initially, MSSA was thought to be a contaminant, but due to the patient’s symptoms, treatment was continued, but antibiotics were de-escalated to cefazolin. Transthoracic echocardiogram showed no valvular vegetations. 

**Figure 1:**
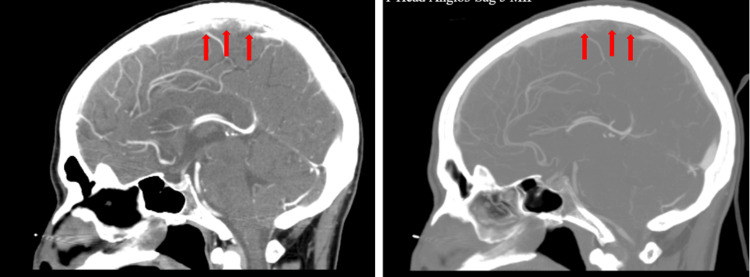
CT venogram showed multiple filling defects (red arrows) suggesting sagittal sinus thrombosis CT: computed tomography

After the de-escalation of antibiotics, the patient had a recurrence of fever, with alterations in the mental status and persistent headache. A lumbar puncture was done, which reflected a picture of bacterial meningitis (see Table 1). Cerebrospinal fluid (CSF) was negative for cultures, syphilis, cryptococcal antigen, herpes simplex 1 and 2, toxoplasma, adenosine deaminase, West Nile virus immunoglobulin M and immunoglobulin G, parvovirus, fungal antibodies, acid-fast bacilli stains and cultures, Lyme line blot, and cysticercus testing. The patient was started on ceftriaxone and acyclovir. A repeat CT scan of the head showed no acute pathology other than an empty sella appearance. Computed tomography (CT) venogram revealed multiple filling defects, suggesting superior sagittal sinus thrombosis (see Figure 1). Therapeutic anticoagulation was started, and the patient’s clinical condition improved significantly, with a resolution of the thrombosis after anticoagulation seen on repeat MR angiography and venogram. The patient was then discharged after the completion of 10 days of ceftriaxone. Thrombotic workups were done, but the parameters were all negative (see Table 2).

**Table 1:** Results of the lumbar puncture WBC: white blood cell; RBC: red blood cell

	Results of the first lumbar puncture	Results of the second lumbar puncture	Results of the lumbar puncture on readmission	Normal value
Opening pressure	29 cm H_2_O	18 cm H_2_O	35 cm H_2_O	6-25 cm H_2_O
Color	Clear	Clear	Clear	Clear
Appearance	Cloudy	Clear	Cloudy	Clear
WBC count	267	40	19,200	<=5.0 cells/uL
Neutrophils %	45	4	1	<=6 %
Lymphocytes %	33	90	94	40-80 %
Monocytes %	13	6	5	15-45 %
RBC count	0	7	356	<=0.0 cells/uL
Protein	185	51	82	15-45 mg/dL
Glucose	29	58	21	40-70 mg/dL

**Table 2:** Results of immunologic and rheumatologic workups IgG- Immunoglobulin G; IgA- Immunoglobulin A, IgM- Immunoglobulin M; DNA- deoxyribonucleic acid

Laboratory exam	Result	Reference range
Antinuclear antibodies (ANA)	Negative	Negative
Anti-dsDNA antibodies (quantitative)	5	0-9 IU/mL
Anti-Smith	<0.2	0-0.9 AI
Sjogren’s anti-SSA	<0.2	0-0.9 AI
Sjogren’s anti-SSB	<0.2	0-0.9 AI
Anti-scleroderma 70 antibodies	<0.2	0-0.9 AI
RNP antibodies	<0.2	0-0.9 AI
Anti-chromatin	<0.2	0-0.9 AI
Anti-Jo 1	<0.2	0-0.9 AI
Anti-centromere B antibodies	<0.2	0-0.9 AI
Anti-U1 RNP (ribonucleoprotein) antibodies	<0.2	<20 units
Complement: C3	167	82-167 mg/dL
Complement: C4	31	12-38 mg/dL
Complement: CH50	>60	>41 U/mL
Anti-cyclic citrullinated peptide (anti-CCP) antibodies, IgG + IgA	<20 units	<20 units
Rheumatoid factor	<10	<14.0 IU/mL
Anti-cardiolipin IgG	<9	0-14 GPL U/mL
Anti-cardiolipin IgM	<9	0-12 MPL U/mL
Anti-cardiolipin IgA	<9	0-11 APL U/mL
Lupus anticoagulant	Negative	Negative
Beta-2 glycoprotein 1 IgG	<9	<21
Beta-2 glycoprotein 1 IgM	<9	<33
Beta-2 glycoprotein 1 IgA	137	<26
Factor II DNA analysis	Negative	Negative
PTT LA (PTT lupus anticoagulant)	34.2	0-43.5 sec
Antithrombin activity	87%	75-135%
Factor V Leiden	Negative	Negative
Protein S- functional	65%	63-140%
Protein C- functional	95%	73-180%

Two weeks after discharge, the patient was brought back to the emergency department for altered mental status and witnessed seizure. The patient again fulfilled the sepsis criteria on admission. Lumbar puncture was repeated, showing a bacterial picture (see Table 1). Blood culture grew *P. luteola*-sensitive to meropenem. The comprehensive ANA panel, hemoglobinopathy evaluations, human immunodeficiency virus (HIV) test, and Quantiferon gold were all negative. The patient was started on meropenem and vancomycin for the infection, and levetiracetam was prescribed as per neurology recommendations. The patient's mental status improved over 48 hours of antibiotic treatment. He was discharged on moxifloxacin 400 mg oral daily for seven days, and his symptoms have resolved.

## Discussion

*P. luteola* case reports have associated this pathogen with septicemia, peritonitis, bacteremia, meningitis, systemic lupus erythematosus, and endocarditis [3,4,5]. However, treatment approaches have been varied, due to the lack of consensus regarding treatment duration and drug of choice.

Several reports of *P. luteola* isolates tested for sensitivity show resistance to ampicillin, tetracyclines, trimethoprim-sulfamethoxazole, and first- and second-generation cephalosporins [3,4]. Meanwhile, susceptibility to third-generation cephalosporins, mezlocillin, imipenem, aminoglycosides, and quinolones is observed [3,4]. The sensitivity profile of our patient’s blood culture study is shown below (see Table 3).

**Table 3:** Antimicrobial sensitivity for Pseudomonas luteola from the patient's blood culture

Antibiotic	Susceptibility
Aztreonam	Resistant
Cefazolin	Resistant
Ceftazidime	Sensitive
Ceftriaxone	Sensitive
Ciprofloxacin	Sensitive
Ertapenem	Sensitive
Gentamicin	Sensitive
Levofloxacin	Sensitive
Piperacillin + Tazobactam	Sensitive
Tobramycin	Sensitive
Trimethoprim + Sulfamethoxazole	Sensitive

Our patient presented with recrudescent meningitis, as he was readmitted within three weeks after treatment. While no organism was isolated during the first admission, *P. luteola* was isolated by a repeat blood culture upon readmission. He had no prosthetic devices, nor any other significant medical history apart from alcohol use disorder. After an extensive literature review, we found only one other case of *P. luteola* meningitis, and it was in an adult with an indwelling prosthetic device [6]. Whether *P. luteola* was the organism causing the first episode of meningitis in our patient remains unclear. 

In addition, clinicians must also be extra vigilant in detecting complications of acute bacterial meningitis, such as cerebral venous sinus thrombosis. Cerebral venous sinus thrombosis (CVST) is a rare but fatal condition that occurs when there is thrombosis of the cerebral veins and dural sinuses [7]. Large sinuses, like the sagittal sinus, are the most commonly involved [8]. The annual incidence of CVT is estimated to be three to four cases per million[9], with a male-to-female ratio of 1.5:5 [10], attributed primarily to prothrombotic states, such as pregnancy, oral contraceptive use, and puerperium [7,8]. Clinical signs and symptoms are often nonspecific and may include headache, benign intracranial hypertension, subarachnoid hemorrhage, focal neurological deficits, seizures, and altered sensorium, which are similar presentations of bacterial meningitis[7,8,11]. Siegel (2023) relates that infection causing the neuroinflammatory response leads to a prothrombotic state, stating that pathogen-associated molecular patterns cause direct damage to the blood-brain barrier and trigger leukocyte reaction [12]. He also adds that cytokines activating the coagulation cascade through the complement system may lead to vasculopathy and hypercoagulation [12].

Thus, it is imperative to educate clinical professionals about* P. luteola* and its implications for pathogenicity, given its potential to induce life-threatening community-acquired and nosocomial infections.

## Conclusions

*P. luteola* is an organism that is rarely a human pathogen but can be fatal if diagnosis is missed. Increased vigilance must always be observed in patients who are immunocompromised or those with an indwelling foreign body. In patients with meningitis suspected of non-improvement or recurrence of symptoms, repeat blood cultures and lumbar punctures are strongly advised if the initial tests showed no organisms or if initial organisms are not responding to treatment. Moreover, further studies are recommended to determine the standard therapy and the duration of treatment for these bacteria. Existing literature leaves much to be explored, as *P. luteola* is not a common organism causing meningitis, and the treatment course is not well defined.
